# Intrathecal Trastuzumab Treatment of the Neoplastic Meningitis due to Breast Cancer: A Case Report and Review of the Literature

**DOI:** 10.1155/2013/154674

**Published:** 2013-01-28

**Authors:** Cristina Dumitrescu, Dominique Lossignol

**Affiliations:** ^1^Department of Medical Oncology, Jules Bordet Institute, Boulevard de Waterloo, 121, 1000 Brussels, Belgium; ^2^Supportive Care Unit, Jules Bordet Institute, Boulevard de Waterloo, 121, 1000 Brussels, Belgium

## Abstract

We report the case of a 65-year-old woman, diagnosed with a breast cancer human epidermal growth factor receptor (HER2) previously negative, who developed leptomeningeal carcinomatosis and was treated with intrathecal (IT) trastuzumab (TST). After five doses of IT trastuzumab, at escalading doses, once weekly, the patient's neurological status stabilised, and that result was maintained for two months. There is evidence in the literature that breast cancer receptor status may change over time, and when it occurs, it may modify the therapeutical approach. We reviewed the pertinent literature and concluded that IT trastuzumab might be a promising treatment for patients with HER2-positive breast cancer leptomeningeal carcinomatosis.

## 1. Case Report

A 65-year-old woman was diagnosed in 1996 to have an invasive ductal breast carcinoma with positive local lymph nodes. Tumor HER2 receptors were negative; estrogen receptor (ER) and progesterone receptor (PgR) were positive. She was treated by a combination of doxorubicin and docetaxel followed by radiotherapy and five years of hormonotherapy with tamoxifen.

From 2004 until August 2011 multiple bone, lymph node, liver, and parenchyma brain metastases were treated with various chemotherapeutic regimens and focal radiation therapy. Brain metastases appeared in August 2008 and were treated by gamma-knife followed by whole brain radiation therapy (WBRT) one month later with partial response. Twenty-eight months later a second course of WBRT was applied for parenchymal progression.

In August 2011 a biopsy of liver metastases revealed the presence of HER2 receptor, contrary to the first diagnostic of breast biopsy which did not showed the presence of the HER2 receptor [1, 2]. On this liver biopsy the status of ER and PgR was not determined.

**Figure 1:**
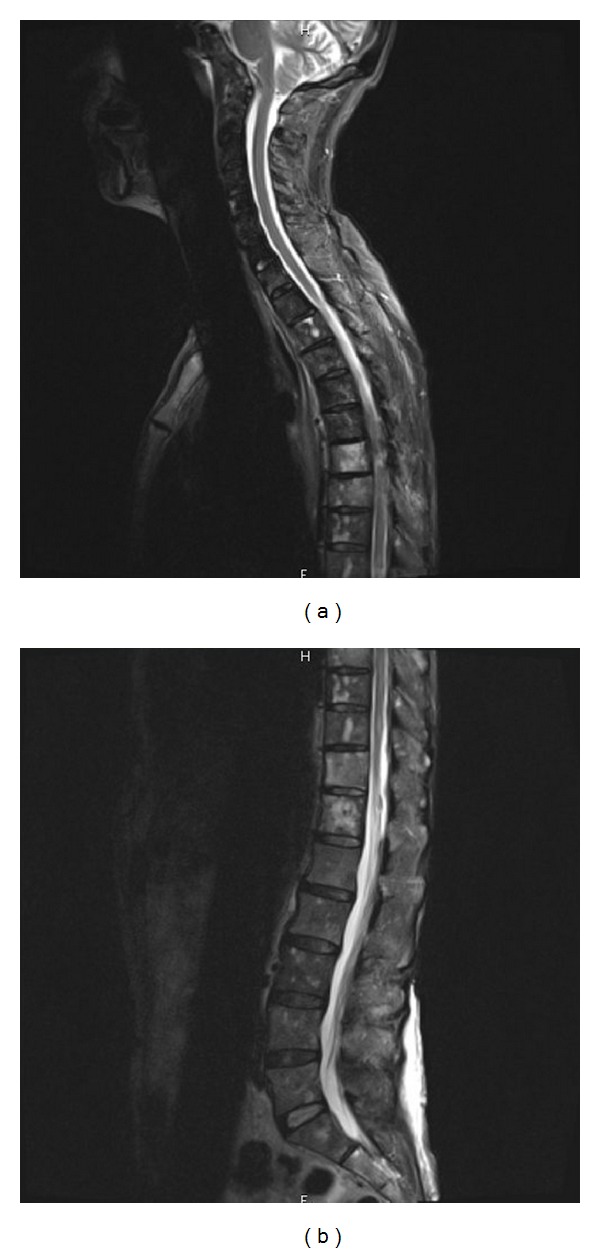
MRI evidence of leptomeningeal carcinomatosis before treatment with IT trastuzumab.

The patient was treated by trastuzumab (4 mg/kg at loading dose, then 2 mg/kg weekly) and vinorelbine. After one cycle, the patient developed ataxia, vertigo, low back pain, headache, and mild cognitive changes. On brain MRI, parenchymal metastatic lesions were stable with no signs of leptomeningeal disease. However spinal MRI showed multiple leptomeningeal lesions (see Figure 1).

A lumbar punction was performed, and the CSF examination showed increased protein level (660 mg/dL), normal glycorrhachia, and the presence of malignant cells.

Intrathecal trastuzumab was given weekly through an Ommaya reservoir, at doses of 20 mg, 40 mg, and then three injections of 100 mg weekly. This treatment was associated with systemic trastuzumab 6 mg/Kg every 3 weeks. Lapatinib treatment was also started but rapidly discontinued because of digestive toxicity.

**Figure 2:**
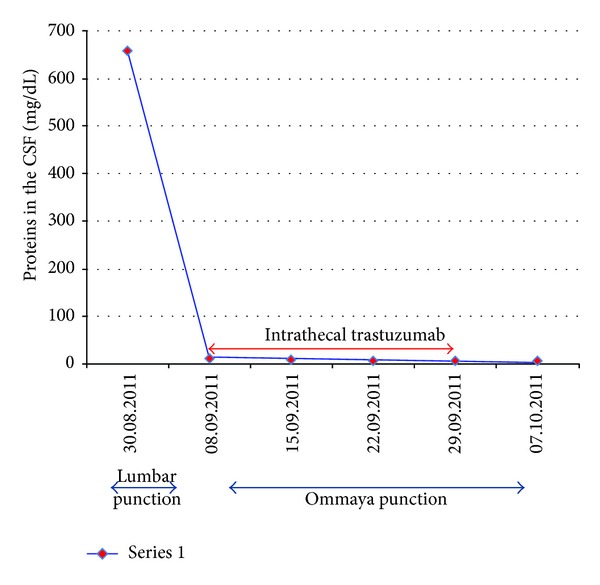
Decreased values until normal ranges of the CSF protein level with IT trastuzumab. First value corresponds to the lumbar punction. The next values corresponds to the Ommaya reservoir functions.

**Figure 3:**
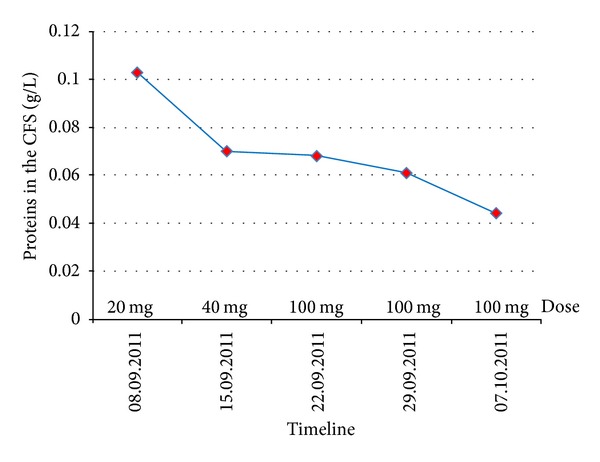
Decreased values of the proteins in the CSF by Ommaya punction, during the treatment with IT trastuzumab.

After the first dose of IT treatment no malignant cells were found in the CSF analysis. The protein level decreased until normal values (0.15–0.45 g/L) after the fourth dose (see Figures 2 and 3).

After the first two doses of intrathecal trastuzumab, the neurological status of the patient stabilised and did not deteriorate further. The MRI showed a reduction of the brain metastatic lesions and a stabilisation of the leptomeningeal infiltration.

The patient died from hepatic failure due to liver metastases eight weeks after the onset of the leptomeningeal disease symptoms.

## 2. Discussion

The first cause of leptomeningeal carcinomatosis is breast cancer (43%), followed by the lung cancer (31%) and melanoma (6%) [9, 10]. Only 5% of meningeal metastasis are diagnosed during the cancer evolution; meanwhile 20% are discovered at the autopsy [11].

Trastuzumab is a murine antibody that recognizes the extracellular domain of the HER2/Neu receptor and has been used successfully for the treatment of breast cancer. But this molecule is unable to penetrate through the blood-brain barrier (BBB) due to its 148000 kiloDa (kD) molecular weight. As usually reported, molecules must not exceed 200 Da to cross the BBB.

In 2000 Pestalozzi and Brignoli [12] measured the concentration of trastuzumab in the CSF in a 62-year-old woman treated with weekly intravenous injections. He demonstrated that a very small amount of the drug penetrates through the BBB: 210 ng/dL in the CSF versus 61392 ng/dL in the serum, 210 minutes after the administration of 120 mg of trastuzumab. In 2007 Stemmler et al. [13] showed that values of CSF trastuzumab were two times higher after radiotherapy and three times higher in presence of leptomeningeal carcinomatosis and radiotherapy [14].

The first treatment using trastuzumab as IT chemotherapy in HER2-positive breast cancer leptomeningeal carcinomatosis was reported by Laufman and Forsthoefel in 2001 [15] in a 48-year-old woman, with no immediate positive therapeutic effect neither any neurological nor site toxicity.

Following this, in 2005 Stemmler et al. [3] highlighted the effectiveness of the IT trastuzumab after using this treatment for a 39-year-old woman with HER2-positive breast cancer who developed headache and dizziness. Five increasing doses, by steps of 5 mg, of IT trastuzumab were administered six months after WBRT. The patient condition improved, and no significant toxicity was observed.

In 2006 Platini et al. [4] published the encouraging results of a 17-month treatment with intrathecal trastuzumab without any clinical adverse events.

A paper published by Colozza et al. [5] described the case of a 38-year-old woman with HER2-positive breast cancer treated with 12.5 mg of IT trastuzumab every 3 weeks. After nineteen months the patient showed no toxicity but had an improved neurological status. A second paper published by Ferrario et al. [6] showed that combined treatment of IT trastuzumab and thiotepa is a promising and safe approach; this conclusion was based on the observation of 31-year-old woman with HER2-positive breast cancer treated with increased doses of IT trastuzumab and then IT trastuzumab with thiotepa (see Table 1).

The hypothesis of using a combined IT treatment was successfully applied by Mego et al. [7] in the case of two HER2-positive breast cancer patients; the treatment consisted in the association of trastuzumab with hydrocortisone and cytarabine. The neurological status improved in both patients, and no malignant cells were further detected in the CSF.

In a paper published by Lombardi et al. [9] in 2011, it is clearly indicated that IT trastuzumab therapy lacks od significant toxicity and can result in significant efficacy compared to other intrathecal drugs (topotecan, etoposide, gemcitabine, *Α* interferon, and alpha interleukin).

Finally in a paper published by Oliveira et al. [8] in 2001, sixty-seven administrations of 25 mg of IT trastuzumab with prednisolone resulted in complete recovery of neurological symptoms in a 40-year-old woman.

Since then trastuzumab had been iteratively used as intrathecal treatment for the HER2-positive breast cancer brain and leptomeningeal metastasis, with no toxicity, increased overall survival, and no chemical interactions [16–19].

There is evidence in the literature [20–24] that tumor receptor status may change over time, and when it occurs, it may modify the therapeutical approach.

In a recent paper published in 2011, Curigliano et al. [25] evaluated the discordance rate of ER, PgR, and HER2 status between primary tumor and liver metastases, with a potential impact on treatment choice. The study was conducted on 255 patients over a ten-year period. Changes in the HER2 status were observed in 172 patients as follows: 17 of 54 patients (31.5%) changed their status from positive to negative and 7 of 118 patients (5.9%) changed their HER2 status from negative to positive. The study also revealed that HER2 status changing from negative to positive was associated with a certain decrease in ER and PgR expression between primary tumor and liver biopsy. Regitnig et al. [22] explained this by a possible crosstalk between the pathways driven by HER2 and the hormone receptors.

Discordance in the receptor status between primary tumor and metastatic site could also be related to different factors: transcriptional and posttranscriptional modification in the gene expression level, that can occur spontaneously or as a consequence of clonal selection in response to chemotherapy, immunotherapy, and hormonal therapy; limited accuracy and reproducibility of receptor assay; errors due to biopsy procedure; real switch in the biology of the disease [25].

Our case report and the review of the literature lead us to conclude that IT administration of trastuzumab might improve or stabilise the consequences of leptomeningeal involvement by HER2-positive breast cancer with no toxicity.

Intrathecal trastuzumab might thus be a promising treatment for leptomeningeal involvement in HER2-positive breast cancer patients, and further perspective studies have to be done to better determine the efficacy, safety, and tolerability of this approach.

## Figures and Tables

**Table 1:** Intrathecal treatments using trastuzumab, alone or in association with other agents.

Reference	Symptoms	WBRT	CSF before ITT		MRI	Intrathecal therapy	CSF after ITT		MRI	Clinical response	Toxicity
		before ITT	Proteins	Malignant C	before ITT		Proteins	Malignant C	after ITT
Stemmler et al., 2006[3]	♀ 39 Y Headache, dizziness	+	Increased	+	No data	MTX 6 × 15 mg + Ommaya **TST 5 mg, 10 mg, 15 mg, 20 mg, 20 mg**	Low	−	No data	Improved	None

Platini et al., 2006 [4]	♀ 36 Y Vertigo, amnesia, mental confusion	−	Increased	+	No data	MTX Peridural PAC **46 × 25 mg TST (17 mo)** THI	Normal	−	No data	Stable	None

Colozzaet al., 2009 [5]	♀ 38 Y Headache, dizziness, gait disorder	+	No lumbar punction		LMC+	Ommaya **23 × 12.5 mg/3** **weeks TST**	No data	−	Stable	Improved	None

Ferrario et al., 2009 [6]	♀ 31 Y Visual impairment, right ptosis, paralysis of the right facial nerve, and left foot drop	−	Normal	+	No data	Ommaya **TST 2 × 20 mg, 2 × 25 mg, 3 × 30 mg, ** **TST 40 mg q3w × 8 mo** **TST 40 mg q3w + thiotepa 10 mg × 6 mo ** **TST 50 mg q3W + 12 mg THI × 13 mo**	No data	−	Improvement	Complete recovery	None

Mego et al., 2011 [7]	♀ 43 Y Dizziness, cranial nerves palsy.	+	Increased	−	LMC+	**MTX 15 mg + CYT 24 mg + HDC 24 mg + 3 × 20 mg TST, 3 × 40 mg TST**	Increased	−	No data	Improved	None
	♀ 39 Y Headache, dizziness, cranial nerve palsies, vision disorders	+	Increased	+	LMC+	**MTX 15 mg + CYT 24 mg + HDC 24 mg + TST 1 × 20 mg, 1 × 40 mg, 1 × 80 mg, 3 × 100 mg**	No data	−		Improved	None

Oliveira et al., 2011 [8]	♀ 40 Y Headache, vomiting, gait disturbance	+	No lumbar punction		LMC+ (CT scan exam)	**TST 25 mg + PRD 25 mg × 25 doses (2 years)**	No data		Partial response	Complete recovery	None

TST: trastuzumab, MTX: methotrexate, THI: thiotepa, HDC: hydrocortisone, PRD: prednisolone, CYT: cytarabine, MRI: magnetic resonance imaging, CSF: cerebrospinal fluid, mo: months, CT: computed tomography, WBRT: whole brain radiotherapy, ITT: intrathecal treatment, “+”: presence, “−”: absence, malignant C: malignant cells.
